# Time interval between alfentanil and rocuronium administration necessary to prevent rocuronium-induced withdrawal movement

**DOI:** 10.1186/s12871-022-01580-1

**Published:** 2022-02-01

**Authors:** Xiao-Dan Wang, Ling-yang Chen, Chun-Lian Zhou, Hai-tao Cong, Nan-jin Chen, Ming-Cang Wang

**Affiliations:** 1 Department of Anesthesiology, Taizhou Hospital of Zhejiang Province Affiliated to Wenzhou Medical University, Zhejiang, China; 2 Department of Anesthesiology, Enze Hospital, Taizhou Enze Medical Center (Group), Zhejiang, China

**Keywords:** Alfentanil, Rocuronium, Pain-induced withdrawal movement

## Abstract

**Background:**

We aimed to determine the time interval between alfentanil and rocuronium administration, at a 50% probability of preventing pain-induced withdrawal movement from rocuronium injection (Time_AR_50).

**Methods:**

A total of 64 patients scheduled for general anesthesia were enrolled in this study (33 men and 31 women). Anesthesia was induced with target-controlled infusion of propofol, at an effect-site target concentration of 3 μg/mL. Then, alfentanil 15 μg/kg was injected for 30 s. After 60 s, rocuronium 0.6 mg/kg was administered to the first patient. The Dixon’s up-and-down method was used to determine the time interval for each subsequent patient (interval of 5 s). Mean arterial pressure (MAP) and heart rate (HR) were recorded at three time points: T0, pre-induction; T1, before rocuronium injection; and T2, 1 min after rocuronium injection.

**Results:**

The Time_AR_50 ± standard deviation (SD) was 5.6 ± 3.7 s and 21.9 ± 5.6 s in the male and female patients, respectively. Based on the probit regression, the Time_AR_50 was 4.7 s (95% confidence interval [CI], 1.2–7.6 s) and 20.3 s (95% CI, 7.7–26.1 s) in the male and female patients, respectively. The Time_AR_95 was 10.6 s (95% CI, 7.7–25.3 s) and 35.0 s (95% CI, 28.1–95.5 s) in the male and female patients, respectively, with significantly higher values in females than in males (*P* < 0.001). Compared with the T0, MAP and HR decreased significantly at T1 and T2 in both groups.

**Conclusion:**

The Time_AR_50 required for preventing rocuronium-induced withdrawal movement were 4.7 s and 20.3 s in male and female patients, respectively.

**Trial registration:**

This study was registered with the Chinese Clinical Trials Registry on April 7, 2021 (URL: http://www.chictr.org.cn. Registry number: ChiCTR2100045137) .

## Background

Rocuronium is an aminosteroidal non-depolarizing neuromuscular blocking agent widely used to induce general anesthesia owing to its muscle relaxation effect. Intravenous rocuronium can cause local burning pain or withdrawal movement in 50–80% of patients, though, more frequently in women [1, 2]. During anesthesia induction, injection pain manifests as involuntary retraction of the injected limb or whole-body movement, which may cause injury, intravenous catheter dislocation, gastric regurgitation, and pulmonary aspiration [3].

Various pharmacological strategies have been used to reduce the incidence and intensity of rocuronium-induced withdrawal movements, such as the use of lidocaine, opioid, and sodium bicarbonate [4–6]. Considering effectiveness and convenience, opioid preconditioning during induction of anesthesia is recommended to reduce rocuronium-induced withdrawal movement [7]. Alfentanil, a narcotic analgesic that stimulates the μ-opioid receptor, has the advantages of rapid onset and short duration. Previous studies showed that alfentanil reduced rocuronium-induced withdrawal movements. Kim et al. showed that alfentanil 10 μg/kg can effectively prevent the pain of rocuronium injection with fewer adverse reactions than remifentanil 1 μg/kg [8]. However, there are no studies on the time interval between alfentanil and rocuronium administration at which there is a 50% probability of preventing pain-induced withdrawal movement from rocuronium injection.

Our study aimed to determine the time interval between the administration of alfentanil and rocuronium administration necessary to prevent rocuronium-induced withdrawal movement in an adult population and to observe the differences between the sexes, so as to provide references for the clinical rational use of the drug.

## Methods

This prospective, double-blind clinical trial study, was approved by the Medical Ethics Committee of Taizhou hospital of Zhejiang Province and registered with the Chinese Clinical Trials Registry on April 7, 2021 (URL: http://www.chictr.org.cn. Registry number: ChiCTR2100045137). Between May 2021 and August 2021, we enrolled 33 male and 31 female patients with American Society of Anesthesiologists (ASA) physical status I or II, aged 20–60 years, and planned to undergo elective day surgery under general anesthesia. Patients with cardiopulmonary disease, drug allergy, asthma, body mass index (BMI) > 28 kg/m^2^ or < 18 kg/m^2^, or those who had received analgesics and sedatives 24 h prior, were excluded from the study. All patients provided written informed consent. They were categorized according to their sex.

All patients fasted from midnight, with no administration pre-induction. Intravenous access was secured with an 18-gauge cannula before entering the operating room. Ringer’s lactate solution was infused. All patients were monitored by electrocardiography, pulse oximetry, non-invasive blood pressure, and end-tidal concentration of carbon dioxide. After pre-oxygenation for 5 min, anesthesia was induced with target-controlled infusion of propofol (Marsh model). The effect-site target concentration of propofol was 3 μg/mL. After the target concentration of propofol was reached, alfentanil 15 μg/kg was injected for 30 s. Once the patient lost consciousness or became apneic, mask ventilation was initiated using 100% oxygen. For each patient, Time_AR_ was defined as the time interval between the end of the alfentanil injection and start of rocuronium injection.

The Time_AR_ was determined by Dixon’s up-and-down method. The first patient in both groups was administered 0.6 mg/kg rocuronium (rocuronium bromide injection, 10 mg/ml, N.V. Organon) at 60 s after the end of the alfentanil injection. The sequential Time_AR_ was increased by 5 s if a patient had a significant movement response to the rocuronium injection or was decreased by 5 s if the response was inhibited. At the time, a nurse injected rocuronium 0.6 mg/kg over 5–10 s and assessed the response. Responsiveness was defined as more than one retraction of the wrist, elbow, and shoulder. The nurse was blinded to the study. The values of Time_AR_ at which there is a 50 and 95% probability of preventing pain-induced withdrawal movement from rocuronium injection were defined as Time_AR_50 and Time_AR_95, respectively.

Mean arterial pressure (MAP) and heart rate (HR) were recorded at the following time points: T0, pre-induction (baseline value); T1, before rocuronium injection; and T2, 1 min after rocuronium injection. The adverse effects of alfentanil, such as hypoxemia, chest wall rigidity, and desaturation were recorded. If the MAP was less than 50 mmHg or less than 20% of the baseline value, phenylephrine (HR > 65 beats/min) or ephedrine (HR < 65 beats/min) was scheduled; if the HR < 50 beats/min, atropine (0.5 mg) was scheduled. At the same time point, the study was terminated and anesthesia was continued according to the judgement of the attending anesthesiologist.

### Statistical methods

Statistical analyses were performed using SPSS (version 23.0; SPSS, Chicago, IL, USA) and Sigma Plot (version 12.5; Systat Software Inc., San Jose, CA). The sample size was calculated according to the Dixon’s up-and-down experimental design [9]. Eight pairs of patients demonstrating “response to injection” and “nonresponse to injection” were collected for statistical analysis by this method. The Time_AR_50 was determined by calculating the mean of the midpoint time of all independent pairs of patients who showed a crossover from “response to injection” to “nonresponse to injection” after eight intersections. For backup analysis, probit regression was used to calculate the Time_AR_50, Time_AR_95, and 95% confidence intervals (CIs).

Data are reported as the mean ± standard deviation (SD) or median (95% CI) or as frequencies (%). Kolmogorov–Smirnov tests were used to test the distribution of continuous data. Patient demographics were analyzed using unpaired t-test or chi-square analysis when appropriate. Hemodynamic data were analyzed by repeated measures analysis of variance, post hoc multiple comparisons were analyzed by Tukey’s test. The Time_AR_50 and Time_AR_95 between groups were compared by a two-sample Z-procedure. Statistical significance was set at *P* < 0.05.

## Results

Data were obtained from 33 male and 31 female adult patients, all of whom completed the study. The patient demographics are presented in Table 1. No significant differences in age, BMI, and ASA physical status were observed between the two groups. None of the patients experienced hypoxemia, chest wall rigidity, or desaturation during the induction of anesthesia.

**Table 1:** Patient demographics

Characteristics	Male	Female
Number of patients(n)	33	31
Age (year)	44.5 ± 14.6	41.8 ± 9.3
Weight (kg)	66.1 ± 8.1	58.4 ± 6.0^*^
Height (cm)	170.6 ± 5.2	158.2 ± 3.8^*^
BMI (kg/m^2^)	22.7 ± 2.4	23.3 ± 2.2
ASA physical status(I/II)	12/21	9/22

The values are expressed as mean ± SD or by the number. *ASA* American Society of Anesthesiologists

^*^P<0.05 compared with the male group

**Fig. 1:**
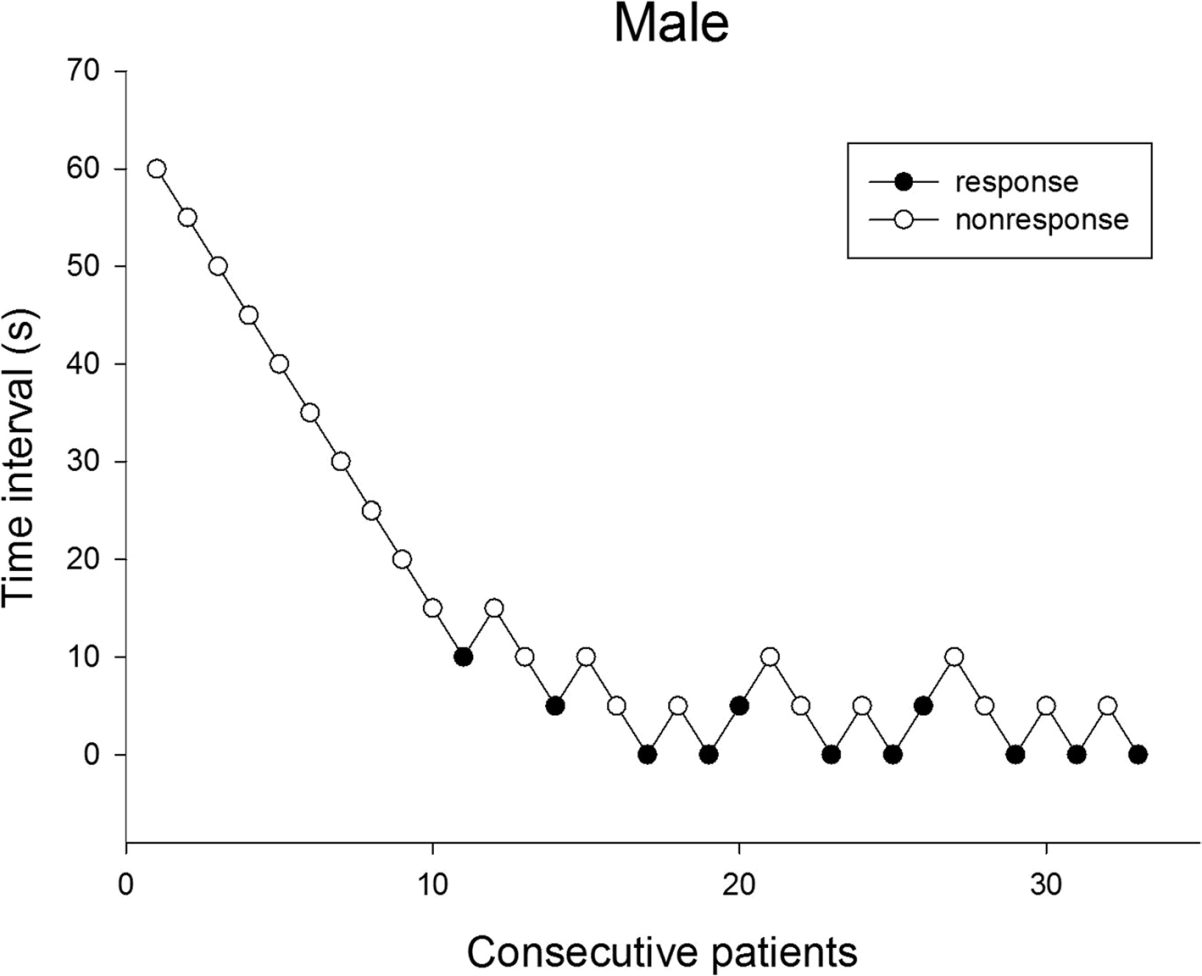
Consecutive time interval and response to rocuronium injection of each male patient

**Fig. 2:**
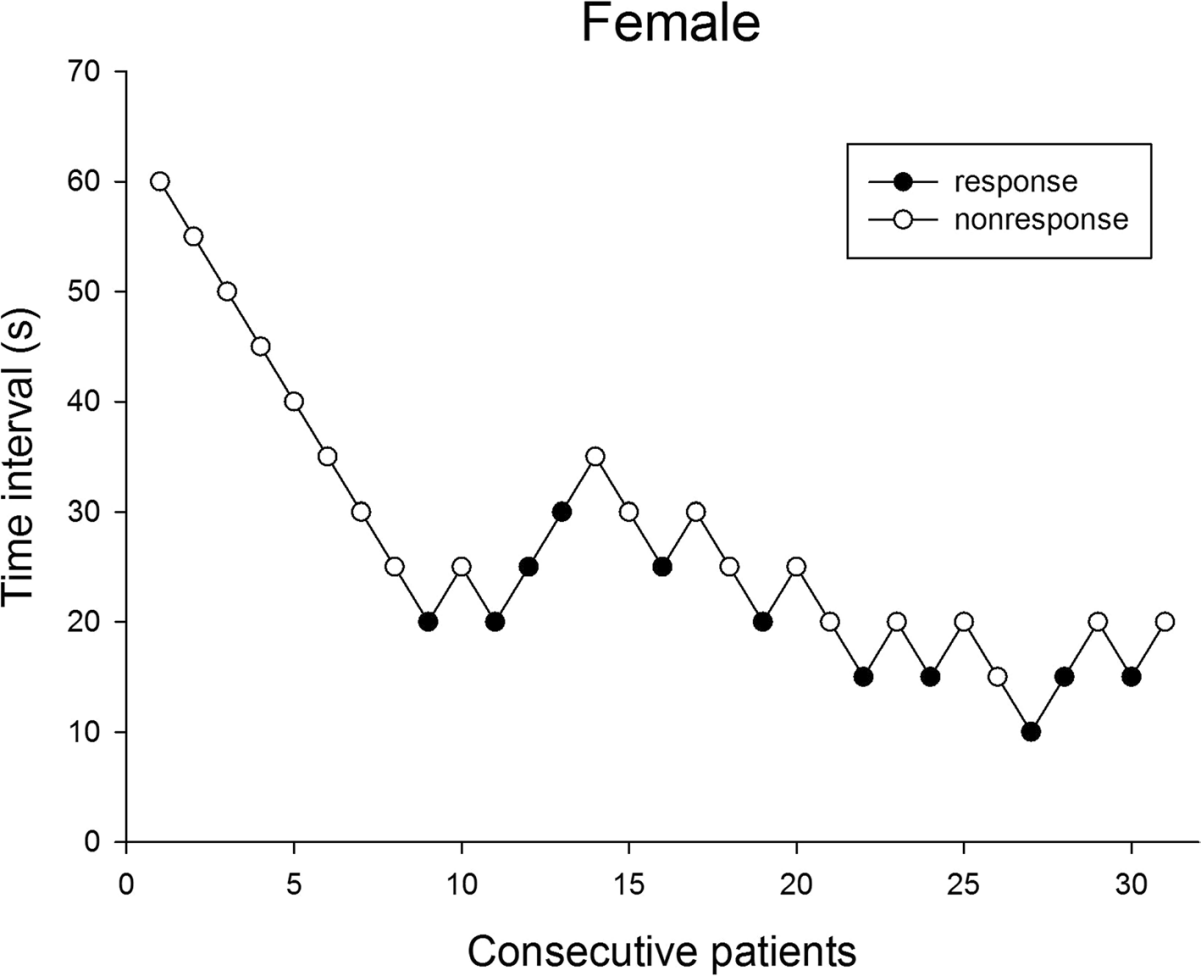
Consecutive time interval and response to rocuronium injection of each female patient

The sequences of the response and nonresponse to rocuronium injection in males and females are shown in Figs. 1 and 2. Using the Dixon’s up-and-down method, the Time_AR_50 was 5.6 ± 3.7 s in the male patients and 21.9 ± 5.6 s in the female patients. In terms of the probit regression, the Time_AR_50 in the male and female patients was 4.7 s (95% CI, 1.2–7.6 s) and 20.3 s (95% CI, 7.7–26.1 s), respectively. The Time_AR_95 in the male and female patients was 10.6 s (95% CI, 7.7–25.3 s) and 35.0 s (95% CI, 28.1–95.5 s), respectively. The Time_AR_95 was significantly longer in females than in males (*P* < 0.001).

The hemodynamic values of both groups of the male and female patients are shown in Table 2. Compared with the baseline value (T0), MAP and HR were significantly decreased at T1 and T2 in both groups. However, none of the patients experienced clinically significant hemodynamic changes during the study period.

**Table 2:** Mean arterial pressure (MAP) and heart rate (HR) during anesthesia induction

		Male(*n* = 33)	Female(*n* = 31)
MAP (mmHg)	T0	100.5 ± 11.7	98.0 ± 8.8
	T1	85.8 ± 8.2^#^	82.0 ± 10.9^#^
	T2	80.6 ± 9.6^#^	81.1 ± 11.1^#^
HR (beat/min)	T0	73.3 ± 13.9	74.8 ± 11.2
	T1	65.8 ± 10.8^#^	66.3 ± 11.3^#^
	T2	62.6 ± 10.8^#^	62.3 ± 8.7^#^

The values are expressed as mean ± SD. T0 on arrival in the operating room (baseline value),T1 before rocuronium injection; T2 1 min after rocuronium injection. ^#^*P*<0.05 compared with T0 within the group

## Discussion

The aim of this study was to determine the appropriate time interval between alfentanil and rocuronium administration needed to prevent pain-induced withdrawal movement from rocuronium injection (Time_AR_).

After loss of consciousness during induction, unexpected reflexes of the wrist, elbow, arm, and shoulder due to the injection of rocuronium can be seen. It has been reported that the incidence of rocuronium-induced withdrawal movement is higher in females than in males [7]; therefore, the participants were categorized into two groups based on their sex. The exact mechanism by which rocuronium induces withdrawal movement has not yet been determined, but it has been reported that it may be due to the osmotic pressure or low pH of the solution directly activating C-nociceptive receptors, or the release of bradykinin, histamine, and other endogenous mediators, as well as substances that mediate inflammation [10]. More recently, generic rocuronium with low glycine concentration has been reported to reduce withdrawal movements compared to the original rocuronium under targeted-controlled infusion of propofol [11].

Due to its fast onset of action, rocuronium is often induced by anesthesia in patients with gastric fullness using non-ventilated techniques, and premature administration induces retraction of the limbs, leading to reflux and aspiration; if the induction time is too long, there is a potential risk of hypoxia. Numerous drug interventions have been reported that can reduce rocuronium-induced withdrawal movements during general anesthesia. Sevoflurane prevented rocuronium-induced withdrawal movement in a time-dependent mode, and the inhalation time of sevoflurane required to prevent withdrawal movement in 50 and 95% of patients were 1.7 and 2.3 min, respectively [12]. The use of opioids during intravenous induction has been widely reported in adults and children to prevent rocuronium-induced withdrawal movement [13]. Ahmad et al. [14] demonstrated that the central analgesic effect of opioids can only occur if sufficient time is allowed to initiate analgesia. Kim et al. [8] demonstrated that remifentanil 1 μg/kg could effectively prevent withdrawal movement in adult patients when administered 90 s before the injection of rocuronium. On the other hand, alfentanil 10 μg/kg was as effective as remifentanil and caused fewer opioid-related adverse reactions. Oh et al. [15] showed that in children, remifentanil, alfentanil, and fentanyl reduced the incidence of rocuronium-induced withdrawal movement. We selected the dose of alfentanil based on Kim et al.’s report [16]., who found that alfentanil 15 μg/kg could be safely administered to prevent rocuronium-induced withdrawal movement and attenuate the increase in MAP and HR after intubation in children. Although alfentanil 15 μg/kg resulted in significant reduction in MAP and HR prior to intubation compared to the baseline in this study, the values were within the normal range and were therefore of little clinical significance.

The occurrence of opioid-related adverse events, such as hypoxemia, chest wall rigidity, and desaturation, during induction of anesthesia is more frequent when administered rapidly than when administered slowly. Therefore, we inject the bolus dose of alfentanil for 30 s. No patient experienced these adverse events during the study. As the peak effect of alfentanil after administration occurs at 1.4 min, a time interval of 60 s between the end of alfentanil injection and the start of rocuronium injection was set in the first case for a maximal effect.

Kim et al. [7] reported that rocuronium-induced withdrawal movement in female patients was 2.1 times that of male patients. Therefore, we observed male and female patients separately to see whether there was also a difference in the time interval between alfentanil and rocuronium administration preventing withdrawal movement induced by rocuronium. Based on the probit regression, the Time_AR_95 in the male and female patients was 10.6 s (95% CI, 7.7–25.3 s) and 35.0 s (95% CI, 28.1–95.5 s), respectively. The Time_AR_95 was significantly longer in females than in males (*P* < 0.001). The Time_AR_95 may be related to the severity of pain. The sex-related difference in the incidence and severity of rocuronium induced pain in adults may be associated with the pain thresholds, pain tolerance levels and sex hormones.

This study has some limitations. First, the confidence intervals of the Time_AR_50 and Time_AR_95 were relatively large. The accuracy of interval estimation may be improved by increasing the sample size, but that is not the purpose of the up-and-down method. The sample size of this study was sufficient for the up-and-down method. To increase the accuracy of the interval estimation, the test space could have been altered in the course of an up-and-down sequence. On the other hand, the injection speed of rocuronium by the nurses (ranging from 5 to 10 s) may have affected the result.

## Conclusion

This study demonstrated that the Time_AR_50 required for preventing rocuronium-induced withdrawal movement was 4.7 s in male and 20.3 in female patients. The Time_AR_95 required for preventing rocuronium-induced withdrawal movement was 10.6 s and 35.0 s in males and females, respectively. The time interval was significantly longer in female group than in male group.

## Data Availability

The datasets generated and analysed during the current study are available from the corresponding author on reasonable request.

## Abbreviations

ASA: American Society of Anesthesiologists
BMI: Body mass index
CI: Confidence intervals
HR: Heart rate
MAP: Mean arterial pressure
